# Supplementary material for: Identification of photoperiod-induced specific miRNAs in the adrenal glands of Sunite sheep (Ovis aries)
Source: Front Vet Sci. 2022 Jul 22;9:888207. doi: 10.3389/fvets.2022.888207 (PMC9354845; doi:10.3389/fvets.2022.888207)
Supplement: Supplementary file 1 [file Data_Sheet_1.ZIP › Supplementary file/miRDeep2 file.zip!/miRDeep2 file/Know_Structure/SP42A1/oar-mir-23b.pdf]

miRBase precursor : oar-mir-23b
Total read count : 13789
oar-miR-23b read count : 13770
remaining reads : 19

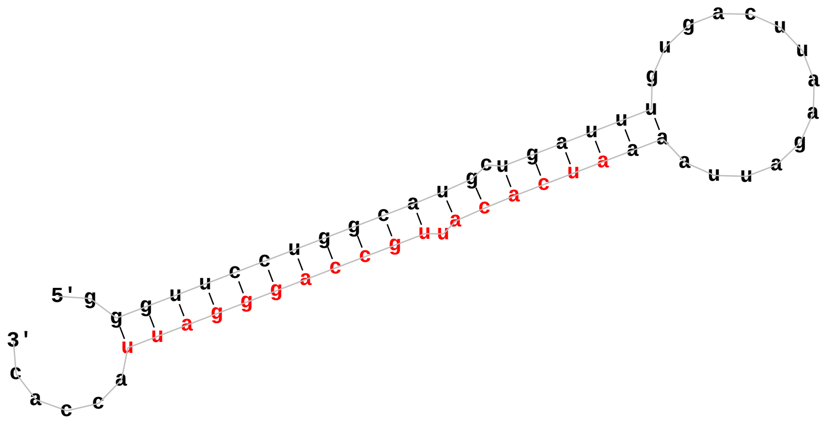

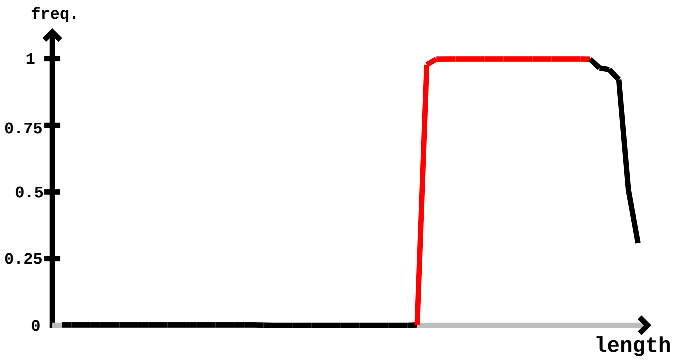


```
                                    oar-miR-23b
5'- ggguuccuggcaugcugauuugugacuuaagauuaaaaucacauugccagggauuaccac   -3'   exp
    ((((((((((((((.((((((..............)))))))).))))))))))..))..    reads      mm         sample
    ggguuccuggcaugcugauu........................................    8          0          seq
    ggguAccuggcaugcugauu........................................    1          1          seq
    ggguuccuggcaugcugauuu.......................................    10         0          seq
    ....................................aaucacauugccagggauu.....    2          0          seq
    ....................................aaucacauugccagggauuac...    1          0          seq
    ....................................aaucacauugccagggauuacc..    11         0          seq
    ....................................aaucacauugccagggauuacca.    3          0          seq
    ....................................aaucacauugccagggauuaccac    2          0          seq
    .....................................aucacauugccagggaC......    1          1          seq
    .....................................aucacauugccagggau......    6          0          seq
    .....................................aucacauugccUgggauu.....    1          1          seq
    .....................................aucacauugccagggauu.....    451        0          seq
    .....................................aucacauugcUagggauu.....    1          1          seq
    .....................................aucacauugcUagggauua....    2          1          seq
    .....................................aucacauugccagggauua....    80         0          seq
    .....................................aucacauugccagggauuac...    506        0          seq
    .....................................aucacauugccagggauuacc..    5600       0          seq
    .....................................aucacauugccagggauuacca.    2698       0          seq
    .....................................aucacauugccagggauuaccac    4109       0          seq
    ......................................ucacauugccagggauu.....    3          0          seq
    ......................................ucacauugccagggauuac...    10         0          seq
    ......................................ucacauugccagggauuacc..    91         0          seq
    ......................................ucacauugccagggauuacca.    46         0          seq
    ......................................ucacauugccagggauuaccac    141        0          seq
    .......................................cacauugccagggauuacc..    1          0          seq
    .......................................cacauugccagggauuacca.    1          0          seq
    .......................................cacauugccagggauuaccac    1          0          seq
    ........................................acauugccagggauuacca.    1          0          seq
    ........................................acauugccagggauuaccac    1          0          seq
```
